# Three-Dimensional Visualization of the Cardiac Stroma

**DOI:** 10.3390/cells14141119

**Published:** 2025-07-21

**Authors:** Florian Kleefeldt, Peter Michelbach, Uwe Rueckschloss, Süleyman Ergün, Nicole Wagner

**Affiliations:** Institute of Anatomy and Cell Biology, Julius-Maximilians-University Würzburg, 97070 Würzburg, Germanysueleyman.erguen@uni-wuerzburg.de (S.E.);

**Keywords:** cardiac stroma, microcirculation, pericytes, serial block-face scanning electron microscopy, 3D reconstruction, telocytes

## Abstract

Cardiac tissue engineering is a promising strategy to restore cardiac function in heart failure patients. Understanding the cardiac tissue architecture including the cardiac stroma is essential for developing not only advanced cardiac tissue engineering but also novel therapeutic strategies. One of the crucial components of the cardiac stroma is the myocardial vasculature. To enhance the spatial visualization of the cardiac stromal cytoarchitecture with a particular focus on myocardial vasculature, we performed 3D reconstructions of the murine cardiac micro vessels using Serial Block-Face Scanning Electron Microscopy (SBF-SEM). These analyses revealed that pericyte cell bodies were primarily oriented lengthwise and extended several cellular protrusions towards the endothelium. At capillary branching points, some pericytes made contact with both capillaries emerging from branching. In addition to pericytes that are completely encapsulated by the common basal lamina together with capillary endothelial cells, we identified other vascular-associated cells located outside this sheath. Based on marker expression, these cells were distinguished from fibroblasts and suggested to be telocytes. The vascular-associated cells formed electron-dense contact zones with endothelial cells, suggesting functional coupling between these both cell types. In conclusion, this study provides detailed three-dimensional visualizations of the cardiac stroma with a particular focus on cardiac microvasculature, offering enhanced insight into the cardiac stromal cytoarchitecture.

## 1. Introduction

Heart failure is a main health issue affecting more than 64 million people globally [1]. Due to donor organ shortage, many people cannot benefit from a necessary cardiac transplantation [2]. Therefore, novel cardiac regeneration or replacement therapies are urgently required. Cardiac tissue engineering using induced pluripotent stem cells (iPSCs) is a promising strategy to solve this problem. Despite advances in cardiomyocyte differentiation from iPSCs, these cells still have an immature fetal-like phenotype compared to adult cardiomyocytes [3].

It is widely recognized that endothelial cells and pericytes as well as stromal cells like telocytes are critically involved in embryonic heart development and proper organ function [4]. However, this was largely neglected in the past. Cardiac tissue engineering mainly focused on cardiomyocyte generation from different sources like iPSCs [5]. Recent evidence suggests that stromal cells also promote maturation of iPSC-derived cardiomyocytes [6,7]. Consequently, more attention should be paid to non-cardiomyocyte cells in tissue engineering applications rather than focusing solely on isolated cardiomyocyte differentiation [8]. Therefore, a better understanding of the regular cytoarchitecture of the mature heart is a prerequisite to improve future iPSC-based cardiac regeneration strategies.

In this study, we analyzed the cardiac microvasculature by 3D reconstruction of murine myocardial capillaries and surrounding stromal tissue using SBF-SEM. This enables an enhanced spatial imagination compared to traditional 2D Transmission Electron Microscopy (TEM). Besides the endothelium and pericytes, we also focused on stromal vascular associated cells (VACs) and potential inter-cellular contacts between these cell types. The resulting 3D reconstructions presented in this study facilitate the understanding of the cytoarchitecture of the cardiac micro vessels with the surrounding stroma in more detail and are therefore of particular interest to all scientists involved in cardiovascular regeneration research and therapy.

## 2. Methods

### 2.1. Mice

C57BL/6J mice (Jackson Laboratory, Bar Harbor, ME, USA) were housed in specific pathogen-free conditions on a 12:12h dark–light cycle and fed standard chow ad libitum. Mice were analyzed at the age of 3 months. All animal experiments were approved by local authorities (Regierung von Unterfranken 55.2-2531.01-68/13) to comply with German animal protection law.

### 2.2. Immunofluorescence of Paraffin Sections

Immunofluorescence analyses were conducted as described previously [9,10]. CD34 antibody was purchased from Abcam (Cambridge, UK; catalogue number: ab8158). CD44 antibody was provided by Biolegend (San Diego, CA, USA; catalogue number: 103002). Primary antibodies were used at a dilution of 1:100. Images were taken using a BZ-X microscope (Keyence, Osaka, Japan).

### 2.3. Serial Block-Face Scanning Electron Microscopy

Mice were euthanized by isoflurane overdose. After thoracotomy, hearts were exposed and perfused with rinsing solution (0.9% NaCl, 0.7% Heparin in ddH_2_0) for 1 min using a perfusion pump (flow rate 4 mL/min) via the left ventricle to remove the intravascular blood. Incision of the right atrium prevented excess intravascular pressure. Perfusion was continued for a further 10 min using a fixative solution (2.5% glutaraldehyde, 2% paraformaldehyde, 2 mM CaCl_2_ in 0.15 M cacodylate buffer pH 7.4). Afterwards, the aorta was perfused retrograde for 1 min to enable proper fixation via coronary arteries. Hearts were then explanted, cut into 1 mm^3^ cubes that were incubated in the fixative solution at 4 °C (O/N). Specimens were washed three times (5 min each) in 0.15 M cacodylate buffer on ice. After incubation in a reduced osmium tetroxide solution (2% osmium tetroxide, 1.5% potassium ferrocyanide, 2 mM CaCl_2_ in 0.15 M cacodylate buffer, pH 7.4) for 1 h on ice, specimens were washed five times (5 min each) in ddH_2_O at room temperature (RT). Following incubation in 1% thiocarbohydrazide/ddH_2_O for 25 min and five washing steps (ddH_2_O), specimens were further incubated in 2% osmium tetroxide/0.5 M cacodylate buffer for 30 min. Then, samples were washed again five times (ddH_2_O) and incubated in aqueous UAR-EMS (4%, Uranyl Acetate Replacement stain; Electron Microscopy Sciences, Hatfield, PA, USA) and stored at 4 °C (O/N). The next day, specimens were washed 3 times (3 min each) in ddH_2_O at RT. Prior to incubation with lead aspartate solution, specimens were washed 2 × 3 min in ddH_2_O at 60 °C and then subjected to en bloc Walton’s lead aspartate staining by incubation in a 0.6% lead aspartate solution according to Walton et al. [11] for 30 min at 60 °C. Afterwards, the solution was slowly cooled to RT. After 5 washing steps (5 min each) in ddH_2_O at RT, specimens were dehydrated in an ice-cold ascending ethanol series (30%, 50%, 70%, 90%, 100%, 100%; 10 min each) followed by acetone solution. Acetone was then replaced by Epon using an ascending series of 25%, 50%, 75% (2 h each), and finally 100% Epon (O/N). After replacement of Epon, specimens were placed in BEEM^®^ capsules and incubated in an oven (60 °C, 48 h) for resin polymerization. The resin-embedded heart fragments were mounted on aluminum pins (Gatan, Pleasanton, CA, USA). Blocks were precision trimmed using a glass knife. Silver paint (Gatan) was used to coat the edges of the tissue block to reduce charging during imaging using BSE mode (variable pressure mode). Analysis of semi-thin sections by TEM was used to identify regions of interest for further analysis and were confirmed in ultra-thin sections prior 3D analysis.

### 2.4. Immunoelectron Microscopy

Vibratome sections (200 μm thickness) of previously fixed (4% PFA in PBS) murine left ventricles (fixation solution: 4% PFA in 0.1 M PBS, pH 7.4) were washed six times (10 min each) with 0.01 M PBS and incubated with 0.5% BSA/TSB (Tris-Saline buffer; 0.01 M; Trizma^®^, 0.1 M NaCl in ddH_2_O, Merck KGaA, Darmstadt, Germany) for 30 min at RT. Afterwards, sections were washed twice (5 min each) with TSB and incubated with the primary antibody (rat anti-CD44, 1:250; rat anti-CD34, 1:250, Merck KGaA, Darmstadt, Germany), diluted in 0.5% BSA/TSB overnight at RT. The primary antibodies described in the immunofluorescence section were also used for immunohistochemistry (1:250 dilution). Subsequently, sections were washed three times in TBS (10 min each) and incubated with the corresponding biotinylated secondary antibody (biotinylated Goat Anti-Rat IgG, 1:500, Merck KGaA, Darmstadt, Germany) for 90 min at RT. Sections were washed in TBS (three times; 10 min each), followed by incubation with avidin-biotinylated peroxidase complex in TBS (Vectastain ABC Kit; Vector Laboratories, Newark, CA, USA; 30 min). Immunolabeling was detected using the glucose oxidase diaminobenzidine method as described previously [12]. After incubation with 2% osmium tetroxide/PBS, specimens were washed twice with ddH_2_O and dehydrated in an ascending ethanol series. First, specimens were incubated in propylene oxide followed by incubation in a 1:1 mixture of propylene oxide and Epon812 O/N. Thereafter, specimens were incubated with pure Epon812 and were finally embedded in Epon812 (48 h at 60 °C). Ultrathin sections (70 nm thick) were cut with an ultramicrotome (Leica Microsystems, Wetzlar, Germany) and collected on copper or nickel grids. Sections were poststained with 2% uranyl acetate/0.2% lead citrate and analyzed with the transmission electron microscope.

### 2.5. Image Acquisition

TEM images were acquired by a LEO 912 AB OMEGA (Carl Zeiss Microscopy, Jena, Germany) electron microscope combined with Image SP Software, Version 1.2.3.46 (x64) for Windows (SysProg & TRS, Moorenweis, Germany). For SBF-SEM, a field emission scanning electron microscope (Sigma 300VP, Carl Zeiss Microscopy GmbH, Munchen, Germany) equipped with an automated ultramicrotome inside the vacuum chamber (3View2XP System; Gatan) was used in combination with DigitalMicrograph-Software Gatan Microscopy Suite^®^ (Version 3.22.1461.0, Gatan). The ultramicrotome cut successive sections at a thickness of 30 nm. After each section, the sample block-face was scanned. The SEM was operated in variable pressure mode (chamber pressure 15 Pa, landing energy 2.2 kV). Immunofluorescent images were captured using a Keyence fluorescence microscope (Keyence, Osaka, Japan; BZ-X series).

### 2.6. Image Processing

Images were aligned using Digital Micrograph (Gatan Microscopy Suite^®^, Gatan, Pleasanton, CA, USA) and saved in DM3 file format. Files were sorted, renamed, and saved using the freeware Rename Us Pro (Version 4.2.3). To convert DM3 files to TIFF, the data were opened in Fiji [13] and saved as an image stack. The contrast of the stack was normalized using the ‘Process’ > ‘Enhance Contrast’ function (saturated pixels set to 1%, with ‘Normalize’ and ‘Process All’ selected). Using the TrakEM2 plugin, a new project was created. The image stack was imported, and user-defined objects (endothelial cells, pericytes, VACs) were defined as area lists in the organizer. The brush tool was used to manually segment each object across each slice or every second slice. After segmentation, area lists were exported as labeled TIFF files and imported into Tomviz (Version 1.8.0, tomviz.org). The background color was set to black, and the color map was manually adjusted by selecting appropriate colors corresponding to the segmented data in the reference bar below the color map. For 3D visualization of the volumetric data, the “Volume” tool was used after adjusting the Z-size. Screenshots and video recordings were created using Captura (Version 8.00). Contrast and brightness were subsequently adjusted in Adobe Photoshop CS4 (Adobe, San José, CA, USA).

## 3. Results

### 3.1. Characterization of Myocardial Stromal Cell by 2D Electron Microscopy

**Figure 1:**
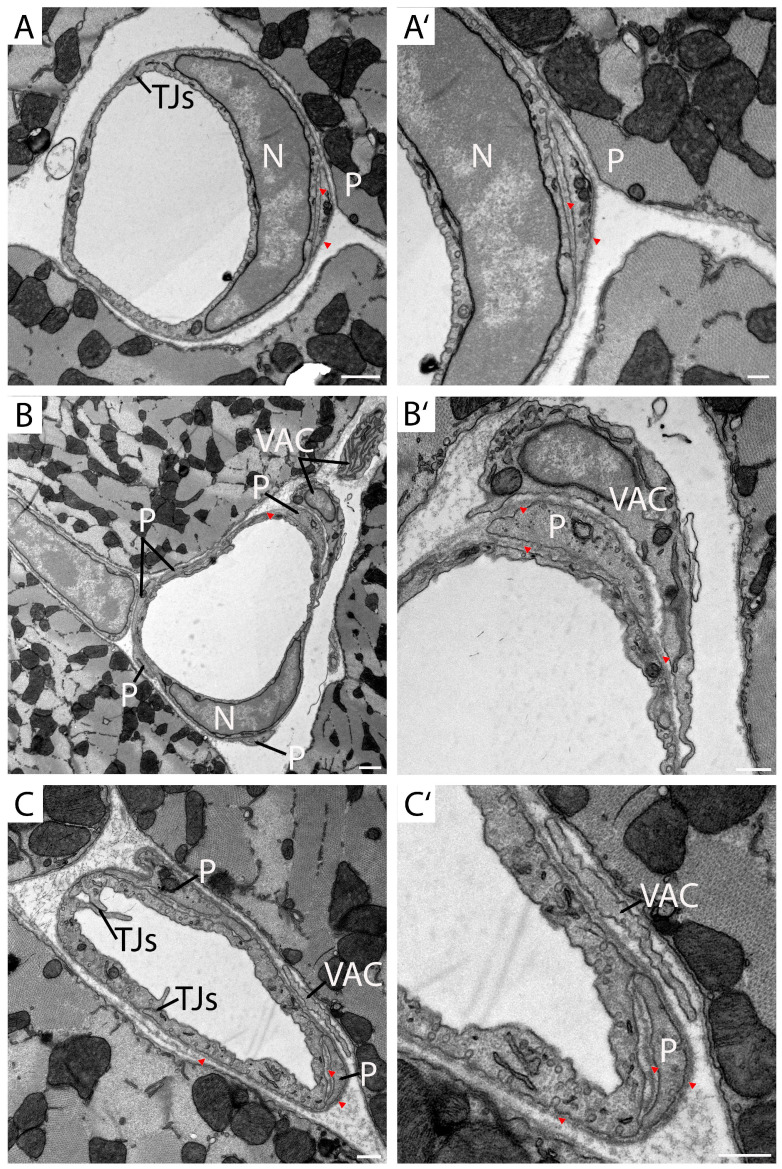
Identification of different cell types by TEM. TEM images (**A**–**C**) and higher magnifications (**A′**–**C′**) of different capillaries in the murine working myocardium (fixation and contrasting using the modified SBF-SEM protocol). N, endothelial cell nucleus. P, pericyte process. VAC, vascular-associated cell process. TJs, tight junctions. Arrow heads, basal lamina. Scale bars: (**A**) 2.5 μm. (**B**,**C**) 1 μm. (**A′**) 250 nm. (**B′**,**C′**) 500 nm.

TEM images showing cross-sections of myocardial capillaries were selected to characterize pericytes and VACs that accompany these capillaries (Figure 1). The capillaries consist of a continuous endothelial cell layer lacking inter-cellular pores or fenestrations. In two capillaries, nuclei of endothelial cells are visible (Figure 1A,A′,B,B′, N). The inter-cellular connections between endothelial cells are indicated by tight junctions (Figure 1A,C, TJs). VAC and pericyte processes are located in proximity to the endothelium (Figure 1 VAC, P). These two cell types can be distinguished from one another due to their different coverage by a basal lamina [14]. In contrast to VACs, pericyte processes are encased by the basal lamina (Figure 1, arrowheads). Both cell types differ from smooth muscle cells that can be found within arterioles, showing a network of contractile filaments (Supplementary Figure S1). For the following 3D reconstructions, different regions of interest (ROIs) were segmented manually, focusing on endothelial cells, pericytes, VACs, and basal lamina.

### 3.2. 3D Reconstruction of ROI 1

**Figure 2:**
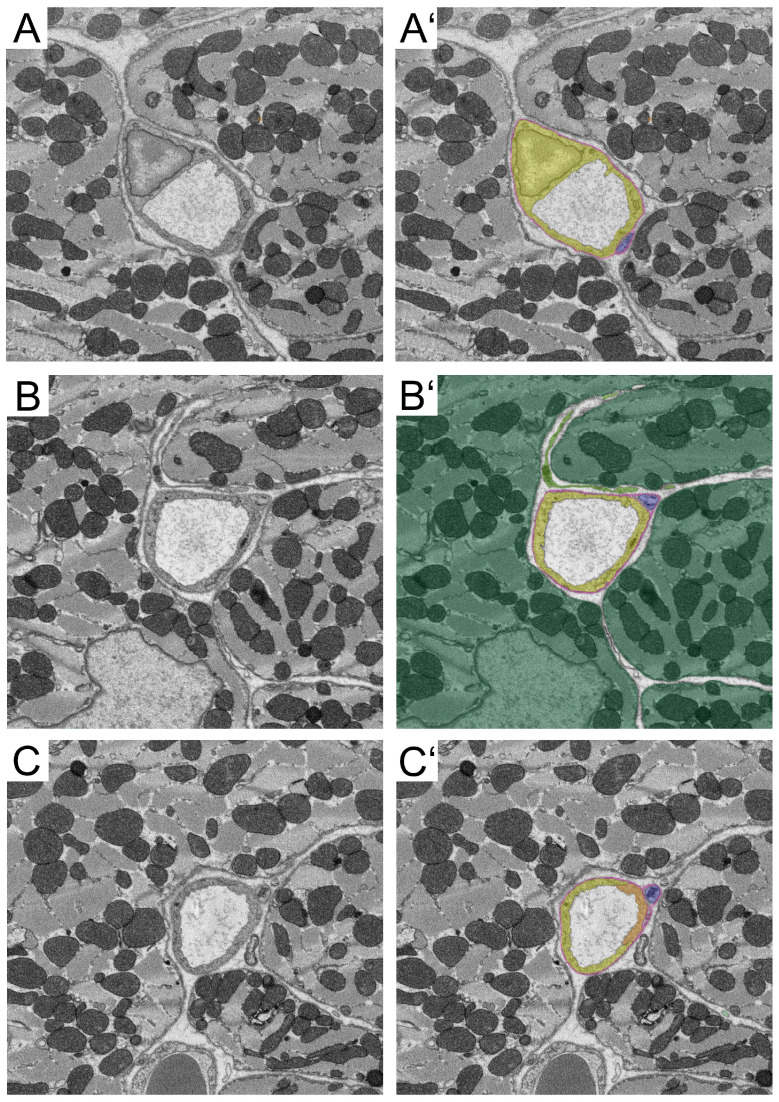
Representative images of ROI 1. Shown are non-segmented images (**A**–**C**) and segmented images (**A′**–**C′**). (**A**,**A′**) section 1/237. (**B**,**B′**) section 103/237. (**C**,**C′**) section 237/237. Segmentation: Yellow, endothelial cell 1. Orange, endothelial cell 2. Magenta, basal lamina. Blue, pericyte. Dark green, vascular-associated cell. Light green, cardiomyocyte. A total of 720 individual images of this capillary were created. For the segmentation, every third image was used.

Representative sections of the capillary (total length: 36.0 µm) of ROI 1 (Figure 2A–C, individual sections; A′–C′ segmented individual sections) showing endothelial cells (Figure 2, depicted in yellow and orange), pericyte processes in close association with the endothelium (Figure 2, depicted in purple) and covered by the continuous basal lamina (Figure 2, depicted in magenta), and one VAC process (Figure 2B,B′, depicted in dark green).

For animation of the complete SBF-SEM data set, please see Supplementary Video S1.

For animation of the segmented SBF-SEM data set, please see Supplementary Video S2.

**Figure 3:**
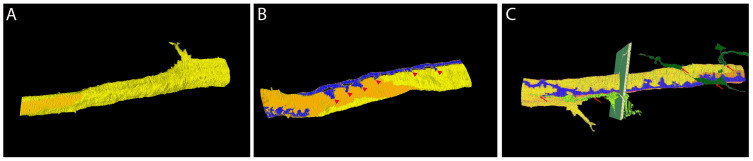
Large volume reconstruction of ROI 1. Three-dimensional reconstruction of endothelial cells (**A**) and pericyte processes (**B**). Three-dimensional reconstruction of endothelial cells, pericyte, and vascular endothelial cell processes (**C**). Yellow, endothelial cell 1. Orange, endothelial cell 2. Blue, pericyte. Dark green, vascular associated cell 1. Light green, vascular associated cell 2. Arrowheads, pericyte extensions approaching the capillary. Arrows, VAC extensions approaching the capillary. Reconstruction: 237 images, 10 nm × 10 nm × 50 nm voxel). For the segmentation, every third image was used.

The 3D reconstruction of the data set of ROI 1 (Figure 3) shows that in this region, the capillary is formed by two different endothelial cells (Figure 3A). The process of one pericyte, running along the axis of the capillary, is closely associated with the endothelium (Figure 3B). It emits smaller branches oriented perpendicularly to the capillary (Figure 3B, arrowheads). The processes of two VACs that extend from the intercellular space between the cardiomyocytes are localized in close proximity of the capillary (Figure 3C). Fine extensions approach the capillary at various sites (Figure 3C arrowheads).

For an animated 3D graphic, please see Supplementary Video S3.

### 3.3. 3D Reconstruction of ROI 2

Representative sections of the capillary (total length: 36.0 µm) of ROI 02 (Supplementary Figure S2A–C, individual sections; A′–C′ segmented individual sections) showing two endothelial cells (Supplementary Figure S2, depicted in yellow and orange), a pericyte in close association with the endothelium (Supplementary Figure S2, depicted in purple) and covered by the continuous basal lamina (Supplementary Figure S2, depicted in magenta), and several processes of VACs (Supplementary Figure S2, depicted in red, rose, green). The VACs have long cellular processes that exceed the analyzed area.

For animation of the complete SBF-SEM data set, please see Supplementary Video S4.

For animation of the segmented SBF-SEM data set, please see Supplementary Video S5.

**Figure 4:**
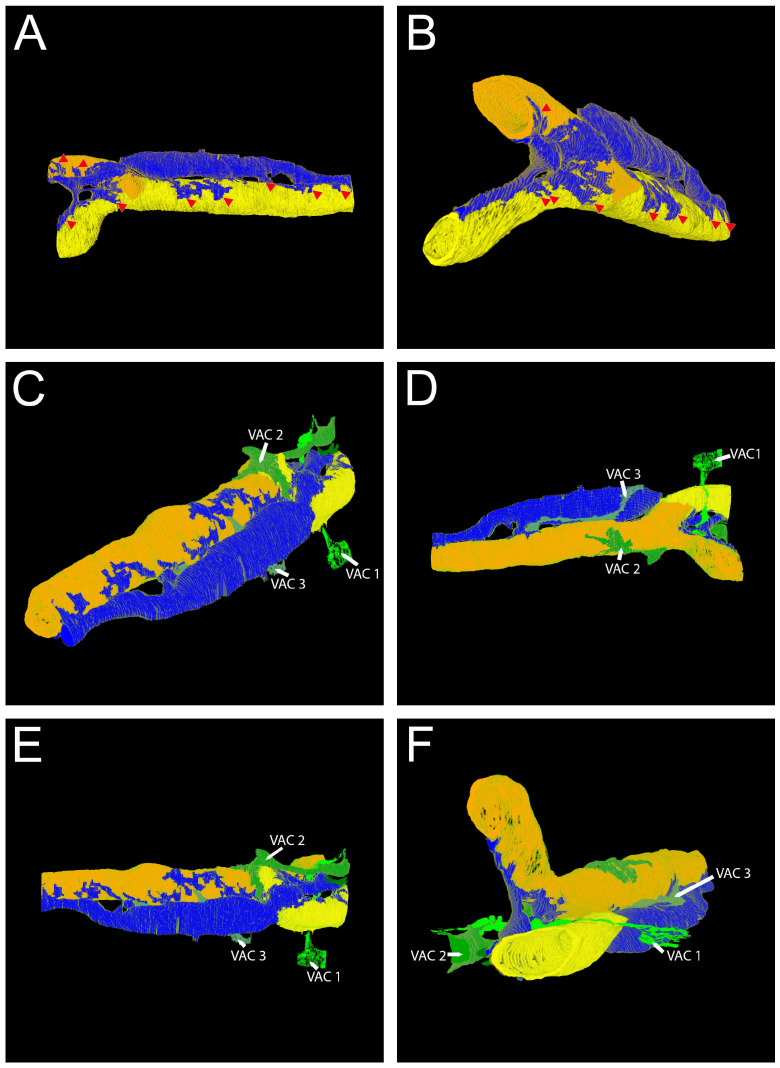
Large volume reconstruction of ROI 2. Three-dimensional reconstruction of endothelial cells (**A**) and pericyte processes (**B**). Three-dimensional reconstruction of endothelial cells, pericyte, and vascular endothelial cell processes (**C**–**F**). The right panels (**B**,**D**,**F**) show alternative views of the respective 3D reconstructions shown on the left (**A**,**C**,**E**). Yellow, endothelial cell 1. Orange, endothelial cell 2. Blue, pericyte. Dark green, vascular-associated cell 1. Light green, vascular-associated cell 2. Pale green, vascular-associated cell 3. Arrowheads, pericyte extensions approaching the capillary. Arrows, VAC extensions approaching the capillary. Reconstruction: 245 images, 10 nm × 10 nm × 50 nm voxel). For the segmentation, every third image was used.

The 3D reconstruction (Figure 4) of the data set shows that the capillary consists of two endothelial cells that form a branching point (Figure 4A,B). The pericyte cell body is located in proximity to the endothelium (Figure 4A,B, depicted in blue) but separated from it by a continuous basal lamina (not depicted in the 3D reconstruction). The processes of the pericyte reach out to both branches of the capillary. Furthermore, VAC processes are present (Figure 4C–F). Neither the 2D nor the 3D model indicate inter-cellular contact between pericytes and the VACs.

For an animated 3D graphic, please see Supplementary Video S6.

### 3.4. 3D Reconstruction of ROI 3

Representative sections of the capillary (total length: 36.7 µm) of ROI 3 (Supplementary Figure S3A–C, individual sections; A′–C′ segmented individual sections) showing two endothelial cells (Supplementary Figure S3, depicted in yellow and orange), two pericytes in close association with the endothelium (Supplementary Figure S3, depicted in light blue and dark blue) and covered by the continuous basal lamina (Supplementary Figure S3, depicted in magenta), and processes of two VACs (Supplementary Figure S3, depicted in light green and dark green). The VACs have long cellular processes which run in the intercellular space that exceed the analyzed area.

For animation of the complete SBF-SEM data set, please see Supplementary Video S7.

For animation of the segmented SBF-SEM data set, please see Supplementary Video S8.

**Figure 5:**
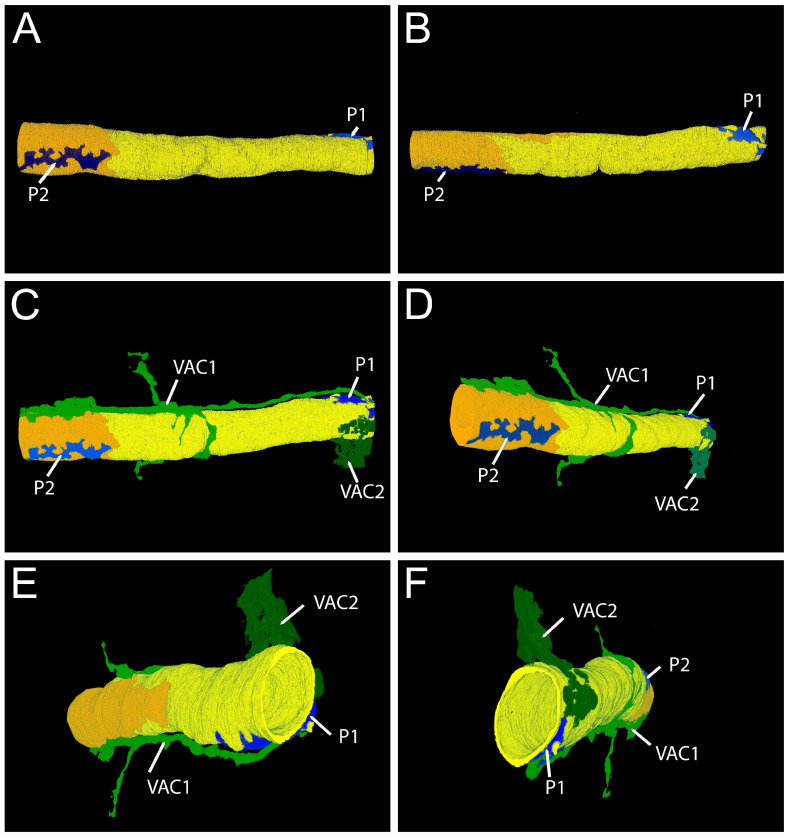
Large volume reconstruction of ROI 3. Three-dimensional reconstruction of endothelial cells and pericyte processes (**A**,**B**). Three-dimensional reconstruction of endothelial cells, pericyte, and vascular endothelial cell processes (**C**–**F**). Yellow, endothelial cell 1. Orange, endothelial cell 2. Light blue, pericyte 1 (P1). Dark blue, pericyte 2 (P2). Light green, vascular-associated cell 1 (VAC1). Dark green, vascular-associated cell 2 (VAC2). Reconstruction: 245 images, 10 nm × 10 nm × 50 nm voxel). For the segmentation, every third image was used.

The 3D reconstruction (Figure 5) of the data set shows that the capillary consists of two endothelial cells (Figure 5A,B). Two pericyte processes, running along the axis of the capillary, are located in proximity to the endothelium (Figure 5A,B) but separated from it by a continuous basal lamina (not depicted in the 3D reconstruction). Several processes of VACs are present (Figure 5E,F). The process of VAC1 is accompanying in the analyzed region, running along the axis of the capillary in very close proximity. In addition, one process of VAC1 runs along the circumference of half of the capillary (Figure 5C,D,F). Neither the 2D nor the 3D model indicate inter-cellular contact between pericytes and the VACs.

For an animated 3D graphic, please see Supplementary Video S9.

### 3.5. 3D Reconstruction of ROI 4

Representative sections of the capillary (total length: 36.7 µm) of ROI 4 (Supplementary Figure S4A–C, individual sections; A′–C′ segmented individual sections) showing two endothelial cells (Supplementary Figure S4, depicted in yellow and orange), one pericyte in close association with the endothelium (Supplementary Figure S4, depicted in blue) and covered by the continuous basal lamina (Supplementary Figure S4, depicted in magenta), and processes of two VACs (Supplementary Figure S4, depicted in light green and dark green). The VACs have long cellular processes, which run in the intercellular space that exceed the analyzed area.

For animation of the complete SBF-SEM data set, please see Supplementary Video S10.

For animation of the segmented SBF-SEM data set, please see Supplementary Video S11.

**Figure 6:**
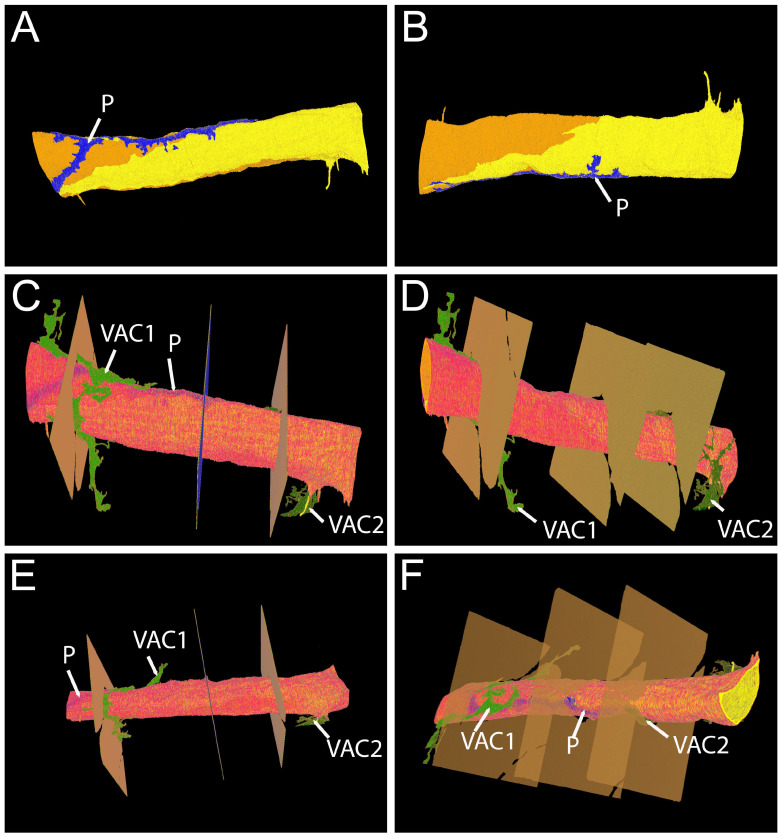
Large volume reconstruction of ROI 4. Three-dimensional reconstruction of endothelial cells and pericyte processes (**A**,**B**). Three-dimensional reconstruction of endothelial cells, pericyte, and vascular endothelial cell processes (**C**,**F**). The right panels (**B**,**D**,**F**) show alternative views of the respective 3D reconstructions shown on the left (**A**,**C**,**E**). Yellow, endothelial cell 1. Orange, endothelial cell 2. Blue, pericyte (P). Light green, vascular associated cell 1 (VAC1). Dark green, vascular associated cell 2 (VAC2). Locations of cardiomyocytes are depicted in brown in 3 individual sections. Reconstruction: 737 images, 10 nm × 10 nm × 50 nm voxel). For the segmentation, every image was used.

The 3D reconstruction (Figure 6) of the data set shows that the capillary consists of two endothelial cells (Figure 6A,B). One pericyte process, running along the axis of the capillary, is located in proximity to the endothelium (Figure 6A,B) but separated from it by a continuous basal lamina (Figure 6 A–D). Several processes of VACs are present (Figure 6E,F). The processes of VAC1 and VAC2 are in close proximity of the capillary. One process of VAC1 runs along the circumference of half of the capillary (Figure 6C,E).

For an animated 3D graphic, please see Supplementary Video S12.

**Figure 7:**
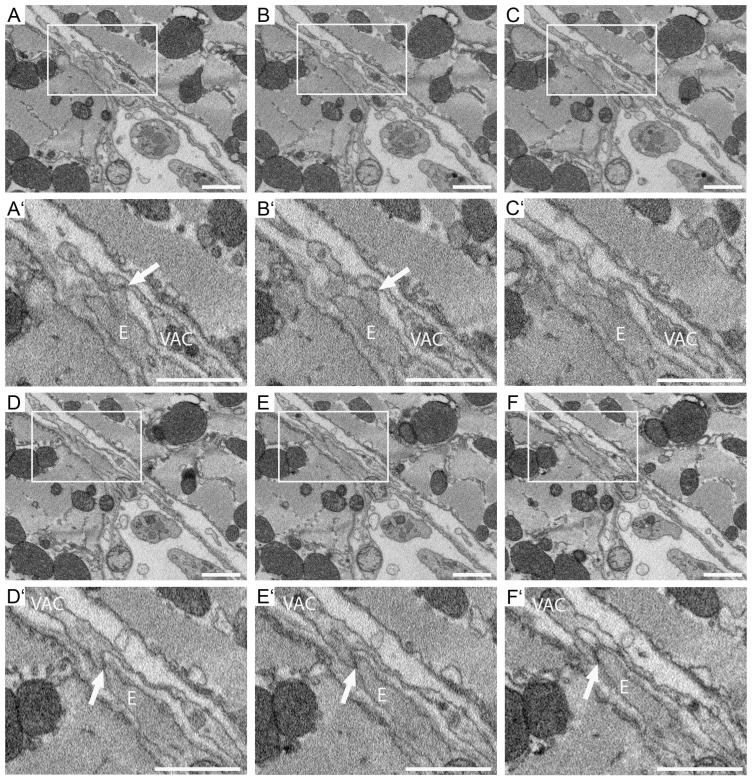
Identification of interaction sites between VACs and endothelial cells in ROI 4. Selected images of the SBF-SEM data set of ROI 4. Shown are non-segmented images (**A**–**F**) and higher magnification of potential VAC-endothelial cell interaction sites (**A′**–**F′**). (**A**,**A′**) section 65/717. (**B**,**B′**) section 66/717. (**C**,**C′**) section 67/717. (**D**,**D′**) section 69/717. (**E**,**E′**) section 70/717. (**F**,**F′**) section 71/717. Potential interaction sites are marked with arrows. E, endothelial cell. VAC, vascular-associated cell process. Scale bars: 1 μm.

Interestingly, two potential interaction sites between endothelial cell 1 and VAC1 were identified (Figure 7). Both lack a basal lamina and show an electron-dense contact area (Figure 7A–F, higher magnifications of the insets in A′–C′ and D′–F′, potential interaction sites are marked with arrows).

### 3.6. 3D Reconstruction of ROI 5

Representative sections of the capillary (total length: 35.8 µm) of ROI 5 (Supplementary Figure S5A–C, individual sections; A′–C′ segmented individual sections) showing four endothelial cells (Supplementary Figure S5, depicted in yellow, beige, orange, light red), two pericytes in close association with the endothelium (Supplementary Figure S5, depicted in light blue and dark blue) and covered by the continuous basal lamina (Supplementary Figure S5, depicted in magenta), and processes of two VACs (Supplementary Figure S5, depicted in light and dark green). The VACs have long cellular processes, which run in the intercellular space that exceed the analyzed area.

For animation of the complete SBF-SEM data set, please see Supplementary Video S13.

For animation of the segmented SBF-SEM data set, please see Supplementary Video S14.

**Figure 8:**
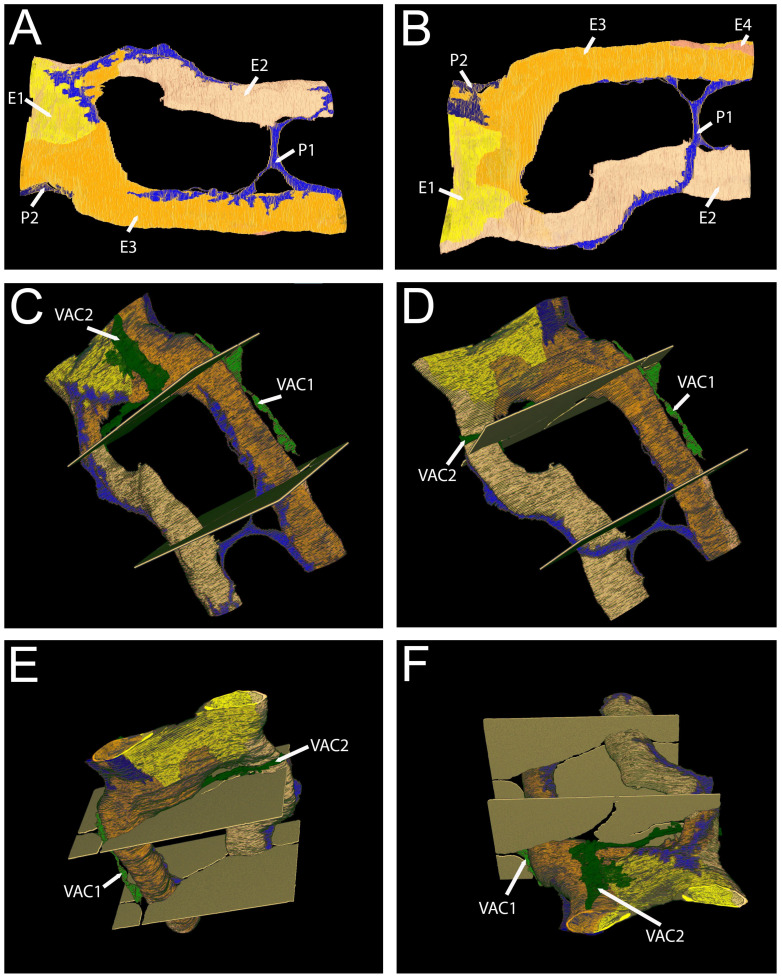
Large volume reconstruction of ROI 05. Three-dimensional reconstruction of endothelial cells (**A**) and pericyte processes (**B**). Three-dimensional reconstruction of endothelial cells, pericyte, and vascular endothelial cell processes (**C**–**F**). The right panels (**B**,**D**,**F**) show alternative views of the respective 3D reconstructions shown on the left (**A**,**C**,**E**). Yellow, endothelial cell 1. Orange, endothelial cell 2. Blue, pericyte. Dark green, vascular associated cell 1. Light green, vascular associated cell 2. Arrowheads, pericyte extensions approaching the capillary. Arrows, VAC extensions approaching the capillary. Reconstruction: 245 images, 10 nm × 10 nm × 50 nm voxel). For the segmentation, every third image was used.

The 3D reconstruction of the data set shows that the capillary this part of the blood vessel is initially composed of two capillaries, which then unite and form a common lumen and later split again into two distinct capillaries (Figure 8A–E). Two pericyte processes, running along the axis of the capillary, are located in close proximity to the endothelium (Figure 8A,B) but separated from it by a continuous basal lamina (not depicted in the 3D reconstruction). The pericyte 1 covers with its processes both parts of the united vessel section and parts of the branched capillaries, running along the axis of the capillary in proximity (Figure 8A,B). The two VACs are located in the immediate vicinity of the united part of the capillary (Figure 8C–F).

For an animated 3D graphic, please see Supplementary Video S15.

**Figure 9:**
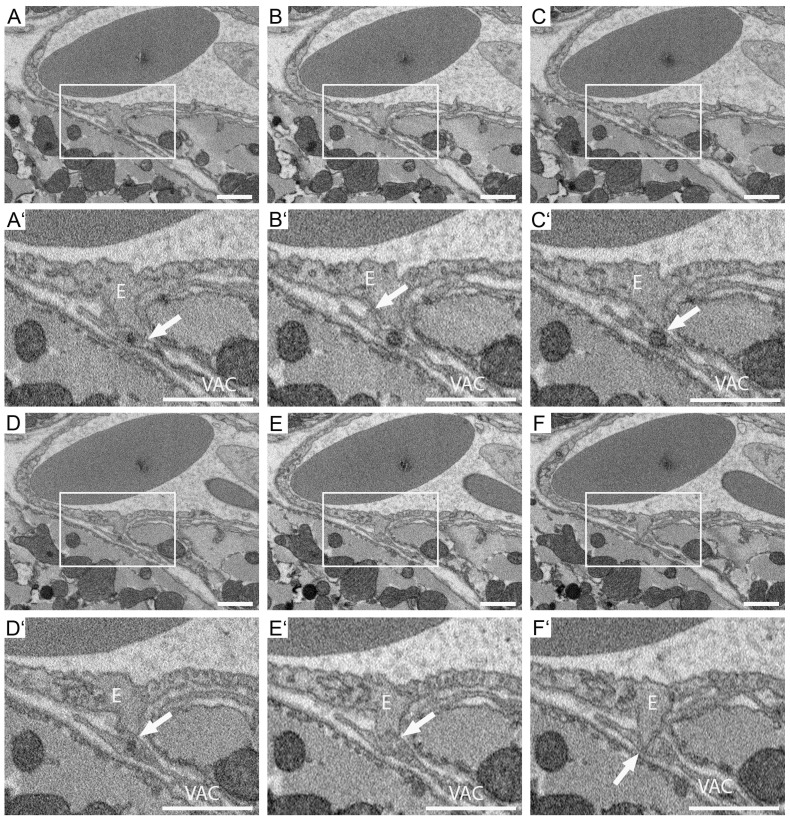
VACs and endothelial cells interaction sites in ROI 5. Selected images of the SBF-SEM data set of ROI 05. Shown are non-segmented images (**A**–**F**) and higher magnification of potential VAC-endothelial cell interaction sites (**A′**–**F′**). (**A**,**A′**) section 86/737. (**B**,**B′**) section 87/737. (**C**,**C′**) section 88/737. (**D**,**D′**) section 90/737. (**E**,**E′**) section 91/737. (**F**,**F′**) section 92/737. Potential interaction sites are marked with arrows. E, endothelial cell. VAC, vascular-associated cell process. Scale bars: 1 μm.

Notably, endothelial cell 2 forms a process that seems to be in contact with VAC 2 (Figure 9). The basal lamina surrounding the endothelial cell is absent in this area, and the potential inter-cellular contact zone appears electron-dense (Figure 9A–F, higher magnifications of the insets in A′–C′ and D′–F′, potential interaction sites are marked with arrows). In this area, the endothelial cell process protrudes towards the VAC (Figure 9A′–F′).

### 3.7. Vascular-Associated Cells Express CD34 and CD44

**Figure 10:**
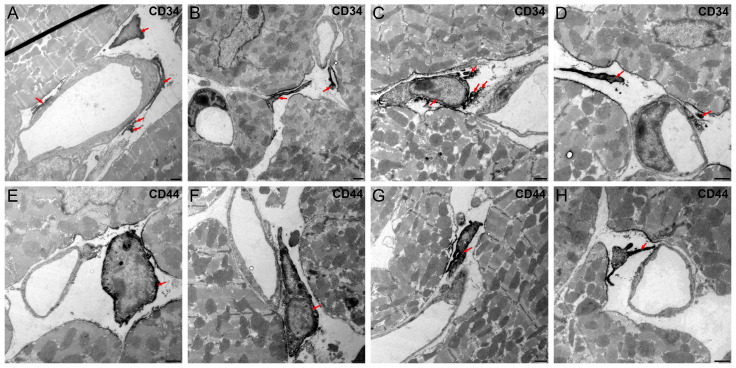
VACs express CD34 and CD44. Detection of CD34 (**A**–**D**) and CD44 (**E**–**H**) at the plasma membrane of VACs by immunoelectron microscopy (red arrows). Scale bars: 1 μm.

Using immunofluorescence staining of paraffin-embedded murine cardiac ventricles, CD34 and CD44 were identified as potential markers of VACs. (Supplementary Figure S6). Immunoelectron microscopy confirmed CD34 and CD44 expression by VACs (Figure 10; corresponding negative control in Supplementary Figure S7). The selection of CD34 and CD44 as markers for vascular-associated cells (VACs) was based on previous reports identifying this combination as characteristic for cardiac stromal cells with telocyte-like features. These cells are morphologically and immunophenotypically distinct from fibroblasts and pericytes, exhibiting long cellular processes and lacking basal lamina coverage. In particular, CD34 has been shown to label both endothelial cells and interstitial stromal cells, while CD44 is a hyaluronic acid receptor associated with stromal cell-matrix interactions and has been used to distinguish telocytes from fibroblasts [15,16,17,18]. The observed co-expression supports the classification of VACs as telocytes.

## 4. Conclusions

Understanding the cytoarchitecture of the heart is a prerequisite in tissue engineering and other cardiac regeneration therapies. Using 3D reconstruction of images generated by SBF-SEM, this study provides detailed insights into the cytoarchitecture of the myocardial microcirculation and stroma, enhancing spatial imagination compared to traditional 2D TEM. The protocol used in this study can also be transferred to other organs or can be applied to study diseases in more detail by comparison of healthy and diseased tissues.

The 3D reconstructions in this study revealed that the cell body of pericytes covering the endothelium of capillaries is predominantly orientated lengthwise. Pericytes can be identified by the continuous basal lamina of the capillary wall that encases both the endothelial cell layer as well as the whole cell body of pericytes including their cellular processes [14]. The 3D reconstructions visualize that these cellular processes only cover a minor portion of the outer endothelial surface. Additionally, in the case of branching capillaries, both branches of the capillary can be reached by a single pericyte. Cellular processes of VACs were detected by the lack of a surrounding basal lamina. These cells extend extensive processes within the cardiac stroma and form contacts that are recognizable by the electron-dense contact zones with endothelial cells at those sites, where the cellular processes of VACs penetrate the endothelial basal lamina. This may suggest a functional coupling between endothelial cells and VACs. The type of contact between endothelial cells and VACs could not be specified based on the acquired images.

VACs differ from cardiac fibroblasts in terms of their morphological shape and expression of CD34 and CD44 [15,16]. Therefore, VACs might represent a cell type called telocyte [19]. These cells were shown to inhibit cardiac microvascular endothelial cell apoptosis after myocardial infarction, to improve angiogenesis and are supposed to influence cardiac development [20,21]. Furthermore, transplantation of telocytes was shown to reduce myocardial infarct size [17]. VACs may influence the endothelium via the inter-cellular contacts detected in our study and thereby may play an important role in maintaining tissue homeostasis. However, this needs to be investigated in more detail in subsequent studies by comparison of healthy and diseased tissues.

Notably, no contact areas between pericytes and VACs were detected, although their cellular processes were located in close proximity. This suggests that VACs and pericytes are not coupled directly. Nevertheless, this does not exclude interaction via paracellular signaling between these cell types.

Taken together, this study provides further insight into the cytoarchitecture of cardiac stroma. Contact areas between VACs and endothelial cells suggest that VACs may influence the cardiac microcirculation. Since VACs were implicated in cardiac development, these cells might also promote maturation and therefore should be considered in cardiac tissue engineering applications [22].

## Data Availability

Dataset available on request from the authors.

## Supplementary Materials

The following supporting information can be downloaded at: https://gigamove.rwth-aachen.de/de/download/d61be61429e55c95583eee4bb1aa3637 (accessed on 10 June 2025), Figure S1: Smooth muscle cell morphology within an arteriole; Figure S2: Selected images of the SBF-SEM data set of ROI 2; Figure S3: Selected images of the SBF-SEM data set of ROI 3; Figure S4: Selected images of the SBF-SEM data set of ROI 4; Figure S5: Selected images of the SBF-SEM data set of ROI 5; Figure S6: Immunofluorescence staining suggests expression of CD34 and CD44 by VACs; Figure S7: Negative control Goat anti-rat IgG; Video S1: SBF-SEM data set of murine cardiac microvasculature (ROI 1); Video S2: SBF-SEM data set of murine cardiac microvasculature (ROI 1); Video S3: Rotational animation of a 3D model of murine cardiac microvasculature (ROI 1); Video S4: SBF-SEM data set of murine cardiac microvasculature (ROI 2); Video S5: SBF-SEM data set of murine cardiac microvasculature (ROI 02); Video S6: Rotational animation of a 3D model of murine cardiac microvasculature (ROI 2); Video S7: SBF-SEM data set of murine cardiac microvasculature (ROI 3); Video S8: SBF-SEM data set of murine cardiac microvasculature (ROI 3); Video S9: Rotational animation of a 3D model of murine cardiac microvasculature (ROI 3); Video S10: SBF-SEM data set of murine cardiac microvasculature (ROI 4); Video S11: SBF-SEM data set of murine cardiac microvasculature (ROI 4); Video S12: Rotational animation of a 3D model of murine cardiac microvasculature (ROI 4); Video S13: SBF-SEM data set of murine cardiac microvasculature (ROI 5); Video S14: SBF-SEM data set of murine cardiac microvasculature (ROI 5); Video S15: Rotational animation of a 3D model of murine cardiac microvasculature (ROI 05).

## References

[1] Savarese G., Becher P.M., Lund L.H., Seferovic P., Rosano G.M.C., Coats A.J.S. (2023). Global burden of heart failure: A comprehensive and updated review of epidemiology. Cardiovasc. Res..

[2] Cameli M., Pastore M.C., Campora A., Lisi M., Mandoli G.E. (2022). Donor shortage in heart transplantation: How can we overcome this challenge?. Front. Cardiovasc. Med..

[3] Schwach V., Passier R. (2019). Native cardiac environment and its impact on engineering cardiac tissue. Biomater. Sci..

[4] Floy M.E., Mateyka T.D., Foreman K.L., Palecek S.P. (2020). Human pluripotent stem cell-derived cardiac stromal cells and their applications in regenerative medicine. Stem Cell Res..

[5] Gisone I., Cecchettini A., Ceccherini E., Persiani E., Morales M.A., Vozzi F. (2022). Cardiac tissue engineering: Multiple approaches and potential applications. Front. Bioeng. Biotechnol..

[6] Lin Z., Garbern J.C., Liu R., Li Q., Mancheño Juncosa E., Elwell H.L.T., Sokol M., Aoyama J., Deumer U.-S., Hsiao E. (2023). Tissue-embedded stretchable nanoelectronics reveal endothelial cell-mediated electrical maturation of human 3D cardiac microtissues. Sci. Adv..

[7] Giacomelli E., Meraviglia V., Campostrini G., Cochrane A., Cao X., van Helden R.W.J., Krotenberg Garcia A., Mircea M., Kostidis S., Davis R.P. (2020). Human-iPSC-Derived Cardiac Stromal Cells Enhance Maturation in 3D Cardiac Microtissues and Reveal Non-cardiomyocyte Contributions to Heart Disease. Cell Stem Cell.

[8] Munawar S., Turnbull I.C. (2021). Cardiac Tissue Engineering: Inclusion of Non-cardiomyocytes for Enhanced Features. Front. Cell Dev. Biol..

[9] Kleefeldt F., Bömmel H., Broede B., Thomsen M., Pfeiffer V., Wörsdörfer P., Karnati S., Wagner N., Rueckschloss U., Ergün S. (2019). Aging-related carcinoembryonic antigen-related cell adhesion molecule 1 signaling promotes vascular dysfunction. Aging Cell.

[10] Götz L., Rueckschloss U., Reimer A., Bömmel H., Beilhack A., Ergün S., Kleefeldt F. (2024). Vascular inflammaging: Endothelial CEACAM1 expression is upregulated by TNF-α via independent activation of NF-κB and β-catenin signaling. Aging Cell.

[11] Walton J. (1979). Lead aspartate, an en bloc contrast stain particularly useful for ultrastructural enzymology. J. Histochem. Cytochem..

[12] Ghavampour S., Kleefeldt F., Bömmel H., Volland J., Paus A., Horst A., Pfeiffer V., Hübner S., Wagner N., Rueckschloss U. (2018). Endothelial barrier function is differentially regulated by CEACAM1-mediated signaling. FASEB J..

[13] Schindelin J., Arganda-Carreras I., Frise E., Kaynig V., Longair M., Pietzsch T., Preibisch S., Rueden C., Saalfeld S., Schmid B. (2012). Fiji: An open-source platform for biological-image analysis. Nat. Methods.

[14] Bonney S.K., Coelho-Santos V., Huang S.-F., Takeno M., Kornfeld J., Keller A., Shih A.Y. (2022). Public Volume Electron Microscopy Data: An Essential Resource to Study the Brain Microvasculature. Front. Cell Dev. Biol..

[15] Sukhacheva T.V., Nizyaeva N.V., Samsonova M.V., Cherniaev A.L., Burov A.A., Iurova M.V., Shchegolev A.I., Serov R.A., Sukhikh G.T. (2021). Morpho-functional changes of cardiac telocytes in isolated atrial amyloidosis in patients with atrial fibrillation. Sci. Rep..

[16] Bei Y., Zhou Q., Fu S., Lv D., Chen P., Chen Y., Wang F., Xiao J. (2015). Cardiac telocytes and fibroblasts in primary culture: Different morphologies and immunophenotypes. PLoS ONE.

[17] Zhao B., Chen S., Liu J., Yuan Z., Qi X., Qin J., Zheng X., Shen X., Yu Y., Qnin T.J. (2013). Cardiac telocytes were decreased during myocardial infarction and their therapeutic effects for ischaemic heart in rat. J. Cell. Mol. Med..

[18] Sukhacheva T.V., Nizyaeva N.V., Samsonova M.V., Chernyaev A.L., Shchegolev A.I., Serov R.A. (2020). Telocytes in the Myocardium of Children with Congenital Heart Disease Tetralogy of Fallot. Bull. Exp. Biol. Med..

[19] Kondo A., Kaestner K.H. (2019). Emerging diverse roles of telocytes. Development.

[20] Cretoiu D., Giuliana Vannucchi M., Bei Y., Manetti M., Faussone-Pellegrini M.S., Ibba-Manneschi L., Xiao J., Maria Cretoiu S., Loewy Z. (2020). Telocytes: New Connecting Devices in the Stromal Space of Organs. Innovations in Cell Research and Therapy.

[21] Liao Z., Chen Y., Duan C., Zhu K., Huang R., Zhao H., Hintze M., Pu Q., Yuan Z., Lv L. (2021). Cardiac telocytes inhibit cardiac microvascular endothelial cell apoptosis through exosomal miRNA-21-5p-targeted cdip1 silencing to improve angiogenesis following myocardial infarction. Theranostics.

[22] Bani D., Formigli L., Gherghiceanu M., Faussone-Pellegrini M.-S. (2010). Telocytes as supporting cells for myocardial tissue organization in developing and adult heart. J. Cell. Mol. Med..

